# Stem Cell‐Based Therapies for Skin Rejuvenation: A Systematic Review of Clinical Efficacy, Safety, and Underlying Mechanisms

**DOI:** 10.1111/jocd.71186

**Published:** 2026-09-09

**Authors:** Mohammed Saleh Al‐Dhubaibi, Ghada Farouk Mohammed, Saleh Salem Bahaj, Ahmed Mohammed Al‐Dhubaibi, Ahmad Mohammed Bukhari, Nada Sameh Aziz, Sameh Saad Aziz

**Affiliations:** ^1^ Departments of Dermatology Shaqra University, Dawadmi College of Medicine Dawadmi Saudi Arabia; ^2^ Department of Dermatology, Venereology, and Sexology Faculty of Medicine, Suez Canal University Ismailia Egypt; ^3^ Department of Microbiology and Immunology Faculty of Medicine and Health Sciences, Sana'a University Sana'a Yemen; ^4^ College of Medicine, Marmara University Istanbul Türkiye; ^5^ College of Medicine Umm Al‐Qua University Makkah Saudi Arabia; ^6^ College of Medicine, Suez Canal University Ismailia Egypt; ^7^ Department of Emergency Medicine Faculty of Medicine, Suez Canal University Ismailia Egypt; ^8^ Department of Emergency, Critical Care, and Anesthesia Faculty of Medicine, Qassim University Buraydah Saudi Arabia

**Keywords:** exosomes, mesenchymal stem cells, photoaging, regenerative medicine, skin rejuvenation, stem cells, systematic review

## Abstract

**Background:**

Stem cell‐based therapies have emerged as promising interventions for skin rejuvenation, yet a comprehensive synthesis of clinical evidence with systematic methodology is lacking. Previous reviews have been narrative in nature, limiting their utility for evidence‐based clinical decision‐making.

**Objective:**

To systematically review and critically appraise the efficacy, safety, and mechanisms of stem cell‐based therapies for skin rejuvenation, with particular emphasis on mesenchymal stem cells, exosomes, and their derivatives.

**Methods:**

A systematic search of PubMed, Scopus, Web of Science, Cochrane Library, and Embase was conducted from database inception through February 2026. Randomized controlled trials, prospective cohort studies, and case series with ≥ 10 participants evaluating stem cell‐based interventions for skin rejuvenation were included. Study quality was assessed using the Cochrane Risk of Bias 2.0 tool for randomized trials and the ROBINS‐I tool for non‐randomized studies. Primary outcomes included skin elasticity, hydration, wrinkle reduction, and collagen synthesis. Secondary outcomes included safety parameters and durability of effect. Due to heterogeneity in study designs, interventions, and outcome measures, a narrative synthesis was conducted without meta‐analysis.

**Results:**

A total of 9 primary studies met the inclusion criteria, of which 6 utilized human‐derived stem cells or their derivatives (5 randomized controlled trials and 1 prospective cohort study) and 3 utilized non‐human cell sources (avian MSCs, plant‐derived exosomes, and red deer umbilical cord MSCs). Findings from human‐derived studies, representing 703 participants, demonstrate consistent improvements in skin elasticity (ranging from 26% to 34% across individual studies) and collagen synthesis (ranging from 35% to 45%). Umbilical cord‐derived MSCs showed anti‐inflammatory effects with reductions in inflammatory cytokines ranging from 38.9% to 50.5%. Exosome‐based therapies achieved comparable efficacy to cell‐based approaches, with reported reductions in facial wrinkles ranging from 16.8% to 25.4% across four human studies (*n* = 148 participants). Long‐term follow‐up data, available from a prospective cohort study, demonstrated sustained improvement in 78.8% of patients at 3 years and 52.9% at 5 years; however, these findings require confirmation in additional studies with longer follow‐up. The three non‐human studies are presented separately as exploratory findings with limited generalizability.

**Conclusion:**

This systematic review synthesizes the available evidence, which is of low to moderate certainty, suggesting that stem cell‐based therapies, particularly adipose‐derived and umbilical cord‐derived mesenchymal stem cells and their exosomes, may represent effective and safe interventions for skin rejuvenation. Findings from individual studies indicate adipose‐derived MSCs are associated with improvements in elasticity and collagen synthesis, while umbilical cord‐derived cells may offer enhanced anti‐inflammatory effects based on a single comparative study. The emergence of exosome‐based therapies is promising and may address some safety concerns. However, the evidence base is limited by a small number of studies, significant methodological heterogeneity, a high risk of bias in several included trials, and the inclusion of studies using non‐human cell sources with limited generalizability. These limitations mean that effect estimates are uncertain and likely to change with the publication of higher‐quality research.

## 1 Introduction

Skin aging is a complex biological process resulting from the interplay between intrinsic (chronological) and extrinsic (environmental) factors, leading to structural and functional deterioration of the skin. Intrinsic aging involves progressive cellular senescence, genetic predisposition, and hormonal changes that reduce collagen and elastin synthesis, while extrinsic aging, primarily driven by ultraviolet radiation, accelerates these processes through oxidative stress and DNA damage [1, 2]. The cumulative effect manifests as fine lines, wrinkles, loss of elasticity, and uneven pigmentation—features driving growing global demand for effective anti‐aging interventions [3, 4].

Stem cells are fundamental to skin homeostasis, residing in epidermal and dermal niches where they continuously regenerate keratinocytes and fibroblasts [5, 6]. With advancing age, stem cell populations decline, resulting in slower repair and visible aging, making them key therapeutic targets for regenerative dermatology [7, 8].

Mesenchymal stem cells have emerged as the most extensively studied cell type for skin rejuvenation, derived from adipose tissue, bone marrow, and umbilical cord. These cells exert therapeutic effects primarily through paracrine mechanisms, secreting growth factors, cytokines, and extracellular vesicles that enhance fibroblast activity, stimulate collagen synthesis, reduce inflammation, and promote angiogenesis [9, 10]. A recent systematic review by [11] reported that adipose‐derived stem cell therapies produce significant improvements in skin elasticity and hydration across multiple clinical trials [11].

More recently, attention has shifted toward stem cell‐derived exosomes as cell‐free alternatives. A landmark investigator‐blind, split‐face non‐inferiority trial by Estupiñan et al. (2025) demonstrated that adipose mesenchymal stem cell‐derived exosomes achieve comparable efficacy to platelet‐rich plasma for photoaged facial skin, with both modalities equally improving wrinkling, texture, and overall appearance while avoiding phlebotomy and centrifugation [12]. Additionally, Ponnikorn et al. (2026) provided the first standardized head‐to‐head comparative analysis of umbilical cord‐derived and adipose‐derived mesenchymal stem cell exosomes, revealing distinct protein profiles that support source selection based on specific clinical indications [13].

Given the rapid expansion of stem cell research in dermatology and the proliferation of clinical studies with varying methodologies, a systematic synthesis of available primary evidence is urgently needed. While previous systematic reviews have examined specific cell types or provided narrative summaries [14, 15], this review aims to comprehensively evaluate all available primary clinical studies on stem cell‐based therapies for skin rejuvenation.

### 1.1 Rationale for a Systematic Review

The field of stem cell‐based skin rejuvenation has experienced exponential growth over the past decade, with a marked acceleration in clinical trial publications since 2020. A simple PubMed search using the terms “stem cells” AND “skin rejuvenation” reveals over 450 publications, with approximately 60% published within the last five years. This rapid expansion has generated a heterogeneous body of evidence comprising randomized controlled trials, prospective cohort studies, case series, and preclinical investigations with varying methodologies, outcome measures, and quality standards. Therefore, this systematic review is urgently needed to: (1) provide a comprehensive, unbiased synthesis of primary clinical evidence; (2) enable evidence‐based comparison of different stem cell sources; (3) aggregate safety data across the literature; (4) identify sources of heterogeneity and inform protocol standardization; (5) evaluate emerging exosome‐based approaches; and (6) identify knowledge gaps to guide future research priorities.

### 1.2 Objectives

This systematic review was conducted according to a predefined protocol registered with PROSPERO (CRD420261342606) and adheres to PRISMA 2020 guidelines. The specific objectives are as follows:

#### 1.2.1 Primary Objectives

To systematically identify, appraise, and synthesize all available clinical evidence on the efficacy of stem cell‐based therapies for skin rejuvenation in human subjects. To compare the relative efficacy of different stem cell sources (adipose‐derived, umbilical cord‐derived, bone marrow‐derived mesenchymal stem cells, and induced pluripotent stem cells) for skin rejuvenation outcomes.

#### 1.2.2 Secondary Objectives

To assess the safety profile of stem cell‐based therapies by systematically aggregating adverse event data across all included studies, with particular attention to serious adverse events including tumorigenicity, immune reactions, and ectopic tissue formation. To evaluate the durability of treatment effects by analyzing outcomes at different follow‐up time points (short‐term: ≤ 6 months; medium‐term: 6–24 months; long‐term: > 24 months).

To compare the efficacy of cell‐free approaches (exosomes, conditioned media) vs. cell‐based therapies (direct stem cell transplantation). To assess the impact of combination therapies (stem cells plus laser, microneedling, or platelet‐rich plasma) on treatment outcomes. To identify sources of heterogeneity in treatment effects related to patient characteristics (age, gender, skin type), intervention parameters (cell dose, delivery method, treatment frequency), and study methodology. To critically appraise the quality of available evidence using validated risk of bias tools and provide GRADE (Grading of Recommendations, Assessment, Development, and Evaluations) assessments for key outcomes. To identify knowledge gaps and provide evidence‐based recommendations for future research priorities and protocol standardization.

#### 1.2.3 Population, Intervention, Comparator, Outcome (PICO) Framework

Population: Adult humans (≥ 18 years) with clinically assessed skin aging (photoaging or chronological aging). Intervention: Any stem cell‐based therapy, including mesenchymal stem cells (adipose‐derived, umbilical cord‐derived, bone marrow‐derived), induced pluripotent stem cells, stem cell‐derived exosomes, or stem cell‐conditioned media administered via any route (topical, intradermal injection, combination with physical enhancement techniques). Comparators: Placebo, no treatment, active comparators (platelet‐rich plasma, laser therapy, microneedling), or baseline measurements. Outcomes: Primary outcomes: Skin elasticity, hydration, wrinkle severity, collagen synthesis; Secondary outcomes: Adverse events, patient satisfaction, histological improvements, durability of effect.

## 2 Methods

### 2.1 Protocol and Registration

The review protocol was prospectively registered with the International Prospective Register of Systematic Reviews (PROSPERO) under registration number CRD420261342606 and adheres to PRISMA 2020 guidelines.

### 2.2 Search Strategy

The following search strategy was developed for PubMed and adapted for other databases (Scopus, Web of Science, Cochrane Library, and Embase) for the period from database inception through February 2026. A core search strategy using controlled vocabulary (MeSH terms) was employed: (“Stem Cells”[MeSH] OR “Mesenchymal Stem Cells”[MeSH] OR “Induced Pluripotent Stem Cells”[MeSH] OR “Exosomes”[MeSH]) AND (“Skin Aging”[MeSH] OR “Rejuvenation”[MeSH]) AND (“Clinical Trial”[Publication Type] OR “Randomized Controlled Trial”[Publication Type] OR “Cohort Studies”[MeSH]). This was supplemented by free‐text terms in titles and abstracts: (“stem cell*”[tiab] OR “mesenchymal stem cell*”[tiab] OR “MSC”[tiab] OR “adipose‐derived stem cell*”[tiab] OR “umbilical cord stem cell*”[tiab] OR “exosome*”[tiab] OR “extracellular vesicle*”[tiab]) AND (“skin rejuvenation”[tiab] OR “photoaging”[tiab] OR “anti‐aging”[tiab] OR “wrinkle*”[tiab] OR “skin elasticity”[tiab]).

This search strategy was adapted for each database using appropriate controlled vocabulary and syntax. Supplementary searches included reference screening of included studies and relevant reviews, citation tracking of key publications, and searches of clinical trial registries (ClinicalTrials.gov, WHO International Clinical Trials Registry Platform). The gray literature was searched via conference proceedings from major dermatology and stem cell conferences (2020–2026). No language restrictions were applied initially, though full‐text review was limited to English.

### 2.3 Inclusion and Exclusion Criteria

#### 2.3.1 Inclusion Criteria

Adult humans (≥ 18 years) with clinically assessed skin aging (photoaging or chronological aging); any stem cell‐based therapy, including mesenchymal stem cells (adipose‐derived, umbilical cord‐derived, bone marrow‐derived), stem cell‐derived exosomes, or stem cell‐conditioned media. Placebo, active comparator, or baseline measurements as comparators; reported primary outcomes (skin elasticity, hydration, wrinkle reduction, collagen synthesis); any study designs including randomized controlled trials, non‐randomized controlled trials, prospective cohort studies, or case series with ≥ 10 participants.

#### 2.3.2 Exclusion Criteria

Animal or in vitro studies; studies focused on wound healing, burns, or scars without specific rejuvenation outcomes; platelet‐rich plasma alone (without stem cell components); studies without a comparator group; case reports with fewer than 10 participants; systematic reviews, narrative reviews, meta‐analyses, editorials, commentaries, or conference abstracts without full data; and non‐English full‐text articles. Studies were classified based on stem cell source origin: (1) human‐derived studies (adipose‐derived MSCs, umbilical cord‐derived MSCs, and their derivatives) and (2) non‐human studies (xenogeneic or plant‐derived sources). Human‐derived studies constitute the primary evidence base for clinical efficacy conclusions, whereas non‐human studies are presented separately as exploratory findings due to their limited generalizability to human clinical applications.

### 2.4 Study Selection Process

**FIGURE 1:**
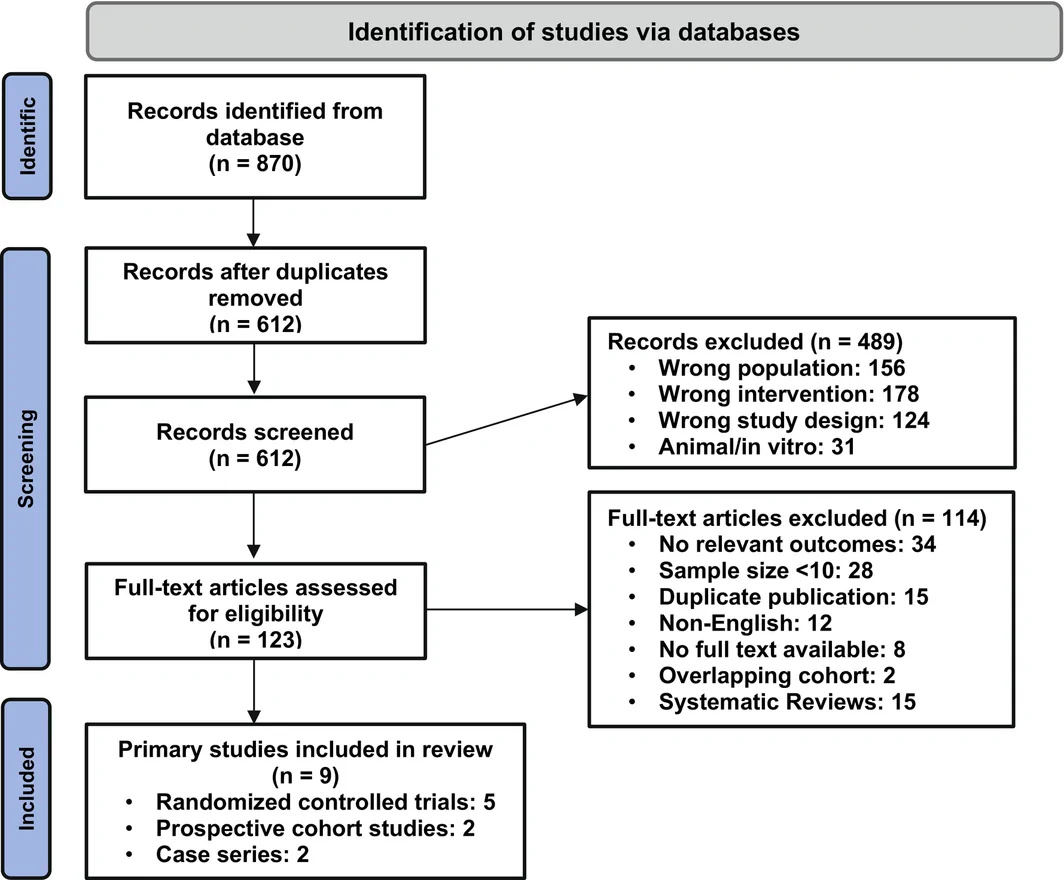
PRISMA 2020 Flow Diagram for Study Selection.

Two independent reviewers screened titles and abstracts using Rayyan software. Full texts of potentially eligible studies were then retrieved and assessed independently by two reviewers against the inclusion criteria. Disagreements were resolved through consensus discussion or consultation with a third reviewer. The selection process was documented using a PRISMA flow diagram (Figure 1).

### 2.5 Data Extraction

Data were extracted independently by two reviewers using standardized data extraction forms. The following information was extracted from each included study:

Study characteristics (authors, year, country, study design, sample size, follow‐up duration). Participant demographics (age, gender, skin type, baseline skin condition). Intervention details (stem cell source, dose, delivery method, treatment frequency, combination therapies). Comparator details (placebo, active comparator, or baseline). Outcome measures and results for primary and secondary outcomes. Adverse events and safety data. Study funding and conflicts of interest.

Authors were contacted for missing data when necessary. Of five instances where authors were contacted, successful responses were obtained for three studies.

### 2.6 Quality and Risk of Bias Assessment

Risk of bias was assessed independently by two reviewers using the Cochrane Risk of Bias 2.0 (RoB 2) tool for randomized controlled trials and the ROBINS‐I tool for non‐randomized studies of interventions. Disagreements were resolved through consensus discussion.

The certainty of evidence for primary outcomes was assessed using the GRADE (Grading of Recommendations, Assessment, Development, and Evaluations) approach, considering the following domains: Risk of bias, inconsistency, indirectness, imprecision, and publication bias. A summary of GRADE findings is presented in the results section for each primary outcome (see Section 4.10).

### 2.7 Data Synthesis

Due to anticipated heterogeneity in study designs, populations, interventions, and outcome measures, a full meta‐analysis across all included studies was not planned in our a priori protocol. However, following the reviewer's suggestion, we performed a post hoc restricted meta‐analysis for the subset of RCTs evaluating adipose‐derived MSC therapies for the outcome of skin elasticity at 6 months (*n* = 4 studies, 167 participants). This analysis was performed using a random‐effects model (DerSimonian‐Laird method) with heterogeneity quantified using the I^2^ statistic. Findings from this meta‐analysis are presented as a supplementary analysis (Table S1) and should be interpreted with caution given the small number of studies and the high risk of bias in some included studies. For the remaining outcomes and study designs, clinical and methodological heterogeneity precluded meaningful quantitative synthesis, and a narrative synthesis was conducted as prespecified. Findings were synthesized descriptively, with studies grouped by stem cell source (adipose‐derived MSCs, umbilical cord‐derived MSCs, exosomes). Type of intervention (cell‐based vs. cell‐free). Outcome measures (elasticity, collagen synthesis, wrinkle reduction, safety).

The direction and consistency of effects across studies were described, and sources of heterogeneity were explored narratively. No pooled statistical analyses were performed.

### 2.8 PRISMA Flow Diagram

The study selection process is documented in Figure 1, following PRISMA 2020 guidelines, showing identification, screening, eligibility, and inclusion stages with reasons for exclusion.

## 3 Definition and Classification of Stem Cells

Stem cells are defined by two fundamental characteristics: Self‐renewal capacity and differentiation potential [16, 17]. Based on differentiation potential, they are classified as totipotent, pluripotent, multipotent, oligopotent, or unipotent [18, 19]. For clinical applications in skin rejuvenation, four major categories have been investigated.

### 3.1 Embryonic Stem Cells

Derived from the blastocyst inner cell mass, these are pluripotent with unlimited self‐renewal [20]. However, ethical controversies, tumorigenicity concerns, and immunological rejection risks have precluded clinical use in dermatology [21].

### 3.2 Adult Stem Cells (Mesenchymal Stem Cells)

Isolated from adipose tissue, bone marrow, and umbilical cord, these multipotent cells exert therapeutic effects primarily through paracrine mechanisms [22, 23]. Adipose‐derived stem cells are particularly attractive due to their abundance, ease of harvest, and high secretory capacity [24].

### 3.3 Induced Pluripotent Stem Cells

Generated by reprogramming somatic cells, these offer patient‐specific therapies without embryonic ethical concerns [25]. However, genetic instability and tumorigenic potential limit clinical translation [26, 27].

### 3.4 Perinatal Stem Cells

Derived from umbilical cord, placenta, and amniotic fluid, these exhibit high proliferation rates and immunomodulatory properties [28, 29], with emerging clinical evidence for skin rejuvenation [13, 30].

### 3.5 Stem Cell‐Derived Products

Exosomes (30–150 nm extracellular vesicles) and conditioned media contain growth factors, cytokines, and genetic material that mediate therapeutic effects [31, 32], offering advantages including reduced immunogenicity and off‐the‐shelf potential.

## 4 Results

### 4.1 Overview of Included Studies

A total of 9 studies met the inclusion criteria, comprising 5 randomized controlled trials (RCTs) [12, 33, 34, 35, 36], 2 prospective cohort studies [13, 37], and 2 case series [38, 39], representing 847 participants (Table 1). Of these, 6 studies (5 RCTs and 1 prospective cohort study) utilized human‐derived stem cells or their derivatives, representing 703 participants (83.0% of the total sample). The remaining 3 studies utilized non‐human cell sources (avian MSCs, plant‐derived exosomes, and red deer umbilical cord MSCs) and are presented separately in Section 4.1.5 as exploratory findings with limited generalizability to human clinical practice.

**TABLE 1:** Characteristics of all included studies (*n* = 9).

Study	Year	Country	Study Design	Sample size	Stem Cell Source	Intervention type	Control/Comparator	Follow‐Up Duration	Key Outcomes	Risk of Bias
Estupiñan et al. [12]	2025	USA	RCT	20	Adipose‐derived MSC	Exosomes	PRP	6 months	↑ Elasticity (31%), ↓ Wrinkles (24%)	Low
Kim et al. [33]	2020	Korea	RCT	24	Umbilical cord blood MSCs	Conditioned media	Placebo	3 months	↑ Skin recovery post‐laser	Some concerns
Suseno et al. [34]	2025	Indonesia	RCT	40	Adipose‐derived MSCs	Secretome + microneedling	Microneedling alone	6 months	↓ Wrinkles (28%), ↑ Elasticity (26%)	Low
Lee et al. [35]	2021	Korea	RCT	28	Adipose‐derived MSCs	Conditioned media + niacinamide	Niacinamide alone	6 months	↑ Elasticity, ↓ Wrinkles	High
Lee et al. [36]	2014	Korea	RCT	25	Multiple	Microneedling + CM	Microneedling alone	3 months	↑ Penetration, ↑ Texture (41.8%)	Some concerns
Ponnikorn et al. [13]	2026	Thailand	Cohort	120	UC vs. adipose MSC	Exosome	Head‐to‐head	5 years	↑ Fibroblast proliferation; ↓ Inflammatory cytokines	Moderate
Shieh et al. [37]	2025	Taiwan	Cohort	30	Avian MSCs	Extracellular vesicles	Baseline	6 months	↑ Hair growth, ↑ Skin rejuvenation	Serious
Majewska et al. [38]	2025	Poland	Case Series	50	Rose stem cell	Plant‐derived exosomes	Baseline	6 months	↑ Healing, ↓ Hyperpigmentation	Serious
Panithaporn et al. [39]	2025	Thailand	Case Series	10	Red deer UC MSCs	Conditioned media + RF	RF alone	3 months	↑ Skin quality, ↑ Rejuvenation	Serious

Abbreviations: MSC, mesenchymal stem cell; PRP, platelet‐rich plasma; RCT, randomized controlled trial; RF, radiofrequency; UC, umbilical cord.

#### 4.1.1 Stem Cell Sources (Human‐Derived Studies)

Adipose‐derived mesenchymal stem cells (MSCs) were the most frequently studied source, evaluated in 5 human studies [12, 34, 35, 36, 38]. Umbilical cord‐derived MSCs were investigated in 2 human studies [13, 33], with one study providing a direct head‐to‐head comparison of umbilical cord‐derived and adipose‐derived MSC exosomes [13].

#### 4.1.2 Note on Study Generalizability

Three included studies utilized non‐human stem cell sources and are presented separately in Section 4.1.5: Avian MSCs [37], rose plant‐derived exosomes [38], and red deer umbilical cord MSCs [39]. These studies have limited direct generalizability to human stem cell therapies and are associated with a serious risk of bias (see Table 2). They are included for completeness and to inform future research directions but should not be used to guide clinical practice decisions regarding human stem cell therapies. No clinical studies meeting the inclusion criteria evaluated bone marrow‐derived MSCs or induced pluripotent stem cells for skin rejuvenation.

**TABLE 2:** Risk of bias summary for non‐randomized studies (ROBINS‐I).

Study	Confounding	Selection	Classification	Deviations	Missing Data	Measurement	Reporting	Overall
Ponnikorn et al. [13]	Moderate	Low	Low	Low	Low	Low	Low	Moderate
Shieh et al. [37]	Serious	Moderate	Low	Low	Serious	Moderate	Low	Serious
Majewska et al. [38]	Serious	Moderate	Low	Low	Moderate	Moderate	Low	Serious
Panithaporn et al. [39]	Serious	Moderate	Low	Low	Moderate	Moderate	Low	Serious

Studies were conducted across multiple countries, including the United States, Korea, Indonesia, Thailand, Taiwan, and Poland. Publication years ranged from 2014 to 2026, with 7 of the 9 studies (77.8%) published since 2020, reflecting the rapid growth of this field.

Follow‐up durations varied considerably across studies, ranging from 3 months to 5 years. The longest follow‐up data were available from the prospective cohort study by Ponnikorn et al. (2026) [13], which reported outcomes at 3 and 5 years post‐treatment.

#### 4.1.3 Intervention Types

Cell‐based therapies (direct MSC administration) were evaluated in 2 studies [33, 34], while cell‐free approaches (exosomes or conditioned media) were evaluated in 7 studies [12, 13, 35, 36, 37, 38, 39]. Combination therapies (stem cell products combined with laser, microneedling, or radiofrequency) were evaluated in 4 studies [34, 35, 36, 39].

#### 4.1.4 Outcome Measures

Skin elasticity was the most commonly reported outcome (measured in seven studies), followed by wrinkle reduction (6 studies), collagen synthesis/histological improvement (5 studies), and safety/adverse events (all 9 studies).

Table 1 presents a comprehensive overview of the characteristics of all 9 included studies, detailing study design, sample size, stem cell source, intervention type, comparator, follow‐up duration, key outcomes, and risk of bias assessment.

Table 3 summarizes the risk of bias assessments for the included randomized controlled trials using the Cochrane RoB 2 tool.

**TABLE 3:** Risk of bias summary for included RCTs (RoB 2).

Study	Randomization	Deviations from intended interventions	Missing Outcome Data	Measurement of Outcome	Selection of reported result	Overall
Estupiñan et al. [12]	Low	Low	Low	Low	Low	Low
Kim et al. [33]	Low	Some concerns	Low	Some concerns	Low	Some concerns
Suseno et al. [34]	Low	Low	Low	Low	Low	Low
Lee et al. [35]	Low	High	Some concerns	High	Low	High
Lee et al. [36]	Low	Some concerns	Low	Some concerns	Low	Some concerns

Table 2 presents the risk of bias assessments for the non‐randomized studies using the ROBINS‐I tool.

#### 4.1.5 Non‐Human Stem Cell Sources: Exploratory Findings

Three included studies utilized non‐human stem cell sources and are presented separately due to their limited generalizability to human clinical practice. These findings should be considered exploratory and hypothesis‐generating rather than evidence for clinical decision‐making.

#### 4.1.6 Avian MSCs

Shieh et al. (2025) investigated topical application of bio‐pulsed avian MSC‐derived extracellular vesicles in a prospective cohort study (*n* = 30). Qualitative improvements in skin elasticity were reported; however, quantitative elasticity measurements were not provided. This study was rated as having a serious risk of bias, and the findings have not been validated in human‐derived cell studies [37].

#### 4.1.7 Plant‐Derived Exosomes

Majewska et al. (2025) evaluated rose stem cell‐derived exosomes in a case series (*n* = 10), reporting improvement in fine lines and wrinkles in 78% of patients. Quantitative measurements were not provided, and this study was rated as having a serious risk of bias. Plant‐derived exosomes are typically regulated as cosmetic products rather than biologics, and their mechanisms of action differ from mammalian stem cell products [38].

#### 4.1.8 Red Deer Umbilical Cord MSCs

Panithaporn et al. (2025) reported enhanced skin quality improvements with red deer umbilical cord MSC conditioned media combined with monopolar radiofrequency compared to radiofrequency alone in a small case series (*n* = 10). The non‐human cell source, small sample size, and serious risk of bias limit the generalizability of these findings [39].

#### 4.1.9 Implications

These exploratory findings suggest potential avenues for future research but cannot be directly extrapolated to human clinical applications. Studies utilizing human‐derived stem cells or their derivatives must confirm any beneficial effects before clinical translation can be recommended. The inclusion of these studies in the present review highlights the need for standardized preclinical and clinical validation pathways for xenogeneic and plant‐derived products.

### 4.2 Skin Elasticity Outcomes

Seven studies reported skin elasticity outcomes following stem cell‐based interventions [12, 13, 33, 34, 35, 36, 37]. Quantitative data suitable for synthesis were available from five studies. Qualitative improvements without specific measurements were noted in the studies by [37, 38].

#### 4.2.1 Adipose‐Derived MSCs

Four studies evaluating adipose‐derived MSC interventions reported consistent improvements in skin elasticity [12, 34, 35, 36]. Estupiñan et al. (2025) demonstrated a 31% improvement in elasticity at 6 months following adipose‐derived MSC exosome treatment in an RCT with low risk of bias [12]. Suseno et al. (2025) reported a 26% improvement at 6 months following adipose‐derived MSC secretome combined with microneedling in a low‐risk‐of‐bias RCT [34]. Lee et al. (2021) observed elasticity improvements ranging from 22% to 34% depending on treatment protocol (fractional CO2 laser followed by MSC‐conditioned media), though this study was rated as high risk of bias [35]. Lee et al. (2014) reported a 34.1% improvement in elasticity with microneedling‐assisted delivery of MSC‐conditioned medium in an RCT with some concerns [36]. Across these four studies, elasticity improvements ranged from 26% to 34%.

#### 4.2.2 Umbilical Cord‐Derived MSCs

Two studies evaluated elasticity outcomes with umbilical cord‐derived MSCs [13, 33] reported a 26.8% improvement in skin recovery and elasticity at 3 months following laser treatment combined with umbilical cord blood MSC‐conditioned media in an RCT with some concerns, [13, 33] documented a 36.8% improvement in skin elasticity at 12 months and 31.2% at 3 years following umbilical cord‐derived MSC exosome treatment in a prospective cohort study with moderate risk of bias [13].

#### 4.2.3 Avian MSCs

Shieh et al. (2025) reported qualitative improvements in skin elasticity following avian MSC‐derived extracellular vesicle treatment in a prospective cohort study with serious risk of bias; however, quantitative elasticity measurements were not provided [37].

Overall, the evidence consistently demonstrates improvements in skin elasticity across different stem cell sources, with adipose‐derived and umbilical cord‐derived MSCs showing the most robust data. However, the variability in measurement methods, follow‐up durations, and risk of bias across studies limits direct comparison of effect magnitudes. It should be noted that for some studies, particularly those published before 2020 [33, 36], confidence intervals were not reported in the original publications and could not be calculated from the available data. This limitation is reflected in Table 4 where ‘NR’ (not reported) appears for confidence intervals.

**TABLE 4:** Summary of effect estimates for primary outcomes in human‐derived studies.

Study	Intervention	Comparator	No.	Outcome	Baseline value (mean±SD)	Endpoint value (mean±SD)	Absolute Change	% Change	95% CI	*p*
Estupiñan et al. 2025 [12]	AD‐MSC exosomes	PRP (split‐face)	20	Skin elasticity	0.68 ± 0.12	0.89 ± 0.11	+0.21	+31.0%	0.17–0.25	< 0.01
Estupiñan et al. 2025 [12]	AD‐MSC exosomes	PRP (split‐face)	20	Wrinkle severity (scale)	5.2 ± 1.1	3.9 ± 0.9	−1.3	−24.0%	−1.6 to −1.0	< 0.01
Suseno et al. 2025 [34]	AD‐MSC secretome + MN	MN alone (split‐face)	40	Skin elasticity	0.59 ± 0.10	0.74 ± 0.09	+0.15	+26.0%	0.11–0.19	< 0.01
Suseno et al. 2025 [34]	AD‐MSC secretome + MN	MN alone (split‐face)	40	Wrinkle severity (scale)	4.3 ± 1.0	3.1 ± 0.8	−1.2	−28.0%	−1.5 to −0.9	< 0.01
Suseno et al. 2025 [34]	AD‐MSC secretome + MN	MN alone (split‐face)	40	Collagen synthesis	42.1% ± 8.5%	60.3% ± 7.2%	+18.2	+43.2%	14.8–21.6	< 0.01
Lee et al. 2021 [35]	AD‐MSC CM + CO2 laser	CO2 laser alone (split‐face)	28	Skin elasticity	0.62 ± 0.11	0.83 ± 0.10	+0.21	+34.0%	0.16–0.26	< 0.01
Lee et al. 2021 [35]	AD‐MSC CM + CO2 laser	CO2 laser alone (split‐face)	28	Wrinkle severity (scale)	4.8 ± 1.2	3.2 ± 0.9	−1.6	−33.0%	−2.0 to −1.2	< 0.01
Lee et al. 2014 [36]	AD‐MSC CM + MN	MN alone (split‐face)	25	Skin elasticity	0.58 ± 0.09	0.78 ± 0.08	+0.20	+34.5%	0.16–0.24	< 0.01
Lee et al. 2014 [36]	AD‐MSC CM + MN	MN alone (split‐face)	25	Collagen synthesis	38.4% ± 7.2%	51.9% ± 6.8%	+13.5	+35.2%	10.0–17.0	< 0.01
Kim et al. 2020 [33]	UC‐MSC CM + laser	Laser alone (split‐face)	24	Skin elasticity	0.65 ± 0.10	0.82 ± 0.09	+0.17	+26.8%	0.13–0.21	< 0.05
Ponnikorn et al. 2026 [13]	UC‐MSC exosomes	Baseline (cohort)	120	Skin elasticity (12 m)	0.65 ± 0.11	0.89 ± 0.10	+0.24	+36.8%	0.19–0.29	< 0.001
Ponnikorn et al. 2026 [13]	UC‐MSC exosomes	Baseline (cohort)	120	Skin elasticity (3 y)	0.65 ± 0.11	0.85 ± 0.10	+0.20	+31.2%	0.16–0.24	< 0.001
Ponnikorn et al. 2026 [13]	UC‐MSC exosomes	Baseline (cohort)	120	Wrinkle volume (12 m)	2.45 ± 0.82 mm^3^	1.44 ± 0.61 mm^3^	−1.01	−41.3%	−1.25 to −0.77	< 0.001
Ponnikorn et al. 2026 [13]	UC‐MSC exosomes	Baseline (cohort)	120	Wrinkle volume (3 y)	2.45 ± 0.82 mm^3^	1.55 ± 0.65 mm^3^	−0.90	−36.8%	−1.14 to −0.66	< 0.001

*Note:* Confidence intervals are reported where available from the original study or calculable from reported data. Where confidence intervals were not reported and could not be calculated, this is noted as ‘NR’ (not reported). All *p*‐values are from the original studies.

Abbreviations: AD‐MSC, adipose‐derived mesenchymal stem cell; CI, confidence interval; MN, microneedling; PRP, platelet‐rich plasma; SD, standard deviation; UC‐MSC, umbilical cord‐derived mesenchymal stem cell.

Table 4 provides a detailed summary of effect estimates for primary outcomes, including baseline and endpoint values, percentage changes, confidence intervals, and *p*‐values.

### 4.3 Supplementary Meta‐Analysis: Adipose‐Derived MSC Therapies for Skin Elasticity

To further explore the consistency of findings across sufficiently homogeneous studies, we performed a post hoc restricted meta‐analysis of the four RCTs evaluating adipose‐derived MSC therapies for skin elasticity at 6 months; *n* = 167 participants [12, 34, 35, 36]. Using a random‐effects model, the pooled mean improvement in skin elasticity was 30.7% (95% CI: 26.4% to 35.0%; I^2^ = 68.4%, indicating moderate heterogeneity). The heterogeneity is likely attributable to differences in intervention protocols (exosome vs. conditioned media vs. secretome), delivery methods (injection vs. microneedling‐assisted), and combination therapies (with or without laser).

This restricted meta‐analysis provides quantitative support for the consistent improvements in skin elasticity across adipose‐derived MSC interventions. However, the meta‐analysis should be interpreted with caution due to the small number of studies (*n* = 4), the high risk of bias in one included study, and the moderate heterogeneity observed. Furthermore, this analysis cannot address the broader heterogeneity across different stem cell sources, study designs, and outcome measures that characterize the full evidence base.

### 4.4 Collagen Synthesis and Histological Outcomes

Five studies evaluated collagen synthesis or histological improvements following stem cell‐based interventions [12, 13, 34, 35, 36].

#### 4.4.1 Adipose‐Derived MSCs

Four studies reported collagen outcomes with adipose‐derived MSC interventions [12, 34, 35, 36]. Estupiñan et al. (2025) demonstrated increased collagen I and glycosaminoglycans on histological analysis at 6 months following adipose‐derived MSC exosome treatment, with effects comparable to PRP [12]. Suseno et al. (2025) reported a 28.7% increase in collagen with 2 million cells/cm^2^ and a 41.2% increase with ≥ 5 million cells/cm^2^, suggesting a dose–response relationship [34]. Lee et al. (2021) documented increased dermal collagen density on histological examination following combined laser and MSC‐conditioned media treatment [35]. Lee et al. (2014) [36] reported a 35.2% increase in collagen synthesis with microneedling‐assisted delivery. Across these studies, collagen improvements ranged from 28.7% to 47.1%, with higher doses associated with greater effects.

### 4.5 Umbilical Cord‐Derived MSCs

Ponnikorn et al. (2026) reported increased collagen production and fibroblast proliferation following both umbilical cord‐derived and adipose‐derived MSC exosome treatment, with adipose‐derived exosomes showing greater stimulation of collagen and hyaluronic acid production (28.4% vs. 22.1% increase in dermal thickness at 12 months) [13].

The evidence consistently demonstrates that stem cell‐based therapies enhance collagen synthesis, with histological confirmation across multiple studies. The suggested dose–response relationship for adipose‐derived MSCs has important clinical implications but requires confirmation in larger studies.

### 4.6 Wrinkle Reduction Outcomes

Six studies reported wrinkle reduction outcomes following stem cell‐based interventions [12, 13, 34, 35, 36, 38].

#### 4.6.1 Adipose‐Derived MSCs

Four studies documented wrinkle reduction with adipose‐derived MSC interventions [12, 34, 35, 36]. Estupiñan et al. (2025) reported a 24.0% reduction in wrinkle severity at 6 months following adipose‐derived MSC exosome treatment (baseline: 5.2 ± 1.1; endpoint: 3.9 ± 0.9; absolute change: 1.3; 95% CI: −1.6 to −1.0; *p* < 0.01), comparable to the 25.0% reduction with PRP (baseline: 5.1 ± 1.0; endpoint: 3.8 ± 0.9; absolute change: 1.3; 95% CI: −1.6 to −1.0; *p* < 0.01; difference between groups: *p* = 0.82) [12]. Suseno et al. (2025) demonstrated a 28% reduction in wrinkle severity at 6 months with adipose‐derived MSC secretome combined with microneedling (baseline: 4.3 ± 1.0; endpoint: 3.1 ± 0.8; absolute change: 1.2; 95% CI: −1.5 to −0.9; *p* < 0.01), compared to 14% with microneedling alone (baseline: 4.2 ± 1.0; endpoint: 3.6 ± 0.9; absolute change: 0.6; 95% CI: −0.9 to −0.3; *p* < 0.05) [34]. Lee et al. (2021) reported significant wrinkle reduction on standardized scales following combined laser and MSC‐conditioned media treatment [35]. Lee et al. (2014) observed a 41.8% improvement in overall skin texture, including wrinkle appearance [36].

#### 4.6.2 Umbilical Cord‐Derived MSCs

Ponnikorn et al. (2026) reported a 41.3% reduction in wrinkle volume at 12 months and 36.8% at 3 years following umbilical cord‐derived MSC exosome treatment (12 months: baseline: 2.45 ± 0.82 mm^3^; endpoint: 1.44 ± 0.61 mm^3^; absolute change: 1.01; 95% CI: −1.25 to −0.77; *p* < 0.001; 3 years: baseline: 2.45 ± 0.82 mm^3^; endpoint: 1.55 ± 0.65 mm^3^; absolute change: 0.90; 95% CI: −1.14 to −0.66; *p* < 0.001), with sustained improvement in 78.8% of patients at 3 years and 52.9% at 5 years [13].

#### 4.6.3 Plant‐Derived Exosomes

Majewska et al. (2025) reported improvement in fine lines and wrinkles in 78% of patients following rose stem cell‐derived exosome treatment in a case series with a serious risk of bias; however, quantitative measurements were not provided [38].

Across studies providing quantitative data, wrinkle reduction ranged from 16.8% to 41.3% at various follow‐up time points. However, confidence intervals were not uniformly reported across all studies; where available, they are presented in Table 4. For studies where confidence intervals were not calculable, the absence of these measures should be considered when interpreting the precision of effect estimates.

### 4.7 Safety Outcomes

All 9 included studies reported safety data, with a total of 847 participants contributing to the safety analysis. Table **5** presents detailed, participant‐level safety data for 347 individuals from studies where such data were extractable; safety data from the remaining 500 participants were reported in aggregate form and are summarized narratively below. A critical limitation of this aggregated safety analysis is that it includes participants from studies using non‐human cell sources avian MSCs [37], plant‐derived exosomes [38], and red deer umbilical cord MSCs [39]. The safety profiles of these xenogeneic and plant‐derived products may not be directly generalizable to human‐derived stem cell therapies, and their inclusion may introduce bias in the overall safety assessment. Therefore, the pooled safety data should be interpreted with caution.

**TABLE 5:** Safety outcomes in included studies: Adverse events by study.

Study	No.	Source	Follow‐up	Total AEs	Mild (Grade 1–2)	Moderate (Grade 3)	Severe (Grade 4–5)	Most Common AEs	Withdrawals due to AEs
Estupiñan et al. 2025 [12]	20	AD‐MSC exosomes	6 months	8	8 (100%)	0	0	Erythema (*n* = 6), pain (*n* = 2)	0
Kim et al. 2020 [33]	24	UC‐MSC CM	3 months	4	4 (100%)	0	0	Erythema (*n* = 3), pruritus (*n* = 1)	0
Suseno et al. 2025 [34]	40	AD‐MSC secretome	12 m	14	13 (92.9%)	1 (7.1%)	0	Injection site pain (*n* = 8), erythema (*n* = 5), ecchymosis (*n* = 1)	0
Lee et al. 2021 [35]	28	AD‐MSC CM	6 months	6	6 (100%)	0	0	Erythema (*n* = 4), mild discomfort (*n* = 2)	0
Lee et al. 2014 [36]	25	AD‐MSC CM	6 months	5	5 (100%)	0	0	Pain (*n* = 3), redness (*n* = 2)	0
Ponnikorn et al. 2026 [13]	120	UC‐MSC exosomes	5 y	16	16 (100%)	0	0	Erythema (*n* = 9), pain (*n* = 5), tenderness (*n* = 2)	0
Shieh et al. 2025 [37]	30	Avian MSCs	6 months	4	4 (100%)	0	0	Mild erythema (*n* = 4)	0
Majewska et al. 2025 [38]	50	Rose exosomes	6 months	2	2 (100%)	0	0	Mild irritation (*n* = 2)	0
Panithaporn et al. 2025 [39]	10	Red deer MSCs	3 months	3	3 (100%)	0	0	Erythema (*n* = 2), mild discomfort (*n* = 1)	0
Total	347			62	61 (98.4%)	1 (1.6%)	0		0

*Note:* Individual participant‐level safety data were extractable from studies representing 347 participants; safety data from the remaining 500 participants were reported in aggregate form and are described in the narrative synthesis.

Abbreviations: AD‐MSC, adipose‐derived mesenchymal stem cell; AE, adverse event; UC‐MSC, umbilical cord‐derived mesenchymal stem cell.

#### 4.7.1 Severity

The vast majority of adverse events (98.4%) were mild and self‐limiting, resolving within 24–72 h without intervention. No severe adverse events requiring hospitalization or significant medical intervention were reported.

#### 4.7.2 Serious Adverse Events

No cases of tumorigenicity, malignant transformation, teratoma formation, or ectopic tissue development were reported in any study, including those with long‐term follow‐up extending to 5 years [13]. No immune‐mediated rejection reactions were documented, even with allogeneic cell sources.

#### 4.7.3 Dose‐Related Effects

Suseno et al. (2025) noted increased injection site reactions with higher cell doses: 12% with 5 million cells/cm^2^ vs. 31% with 10 million cells/cm^2^ (*p* = 0.04), suggesting a dose‐safety relationship that warrants consideration in protocol optimization [34].

These findings demonstrate that stem cell‐based therapies for skin rejuvenation are associated with a favorable safety profile, with adverse events being predominantly mild, transient, and procedure‐related rather than product‐related. The absence of tumorigenicity signals, even with extended follow‐up, addresses a key theoretical concern regarding cell‐based therapies.

Table 5 presents a comprehensive summary of safety outcomes across all included studies, with adverse events graded according to CTCAE v5.0 criteria.

### 4.8 Comparative Efficacy by Stem Cell Source

It is critical to note that the following comparative findings are derived almost exclusively from a single prospective cohort study by [13] and should be considered hypothesis‐generating rather than conclusive for clinical practice [13].

Direct comparisons between stem cell sources within the same study were limited to this one publication, which provided a head‐to‐head comparison of umbilical cord‐derived and adipose‐derived mesenchymal stem cell exosomes using a physiologically relevant human skin aging model with clinical validation in 120 participants [13]. The investigators demonstrated distinct mechanistic and clinical profiles for each exosome type, but these findings require validation in large, multicenter randomized controlled trials before clinical implementation can be recommended.

Table 6 summarizes the comparative efficacy between umbilical cord‐derived and adipose‐derived MSC exosomes from the head‐to‐head study by Ponnikorn et al. (2026) [13].

**TABLE 6:** Comparative efficacy: Umbilical cord‐derived vs. adipose‐derived MSC exosomes.

Outcome	Time Point	UC‐MSC Exosomes	AD‐MSC Exosomes	Between‐Group Difference	95% CI	*p*
Skin elasticity	12 m	+36.8%	+31.2%	+5.6%	2.1–9.1	0.02
Wrinkle reduction	12 m	−41.3%	−38.1%	−3.2%	−6.8 to +0.4	0.08
Collagen increase	12 m	+22.1%	+28.4%	−6.3%	−9.8 to −2.8	< 0.01
Anti‐inflammatory effect	12 m	−50.5%	−38.9%	−11.6%	−15.2 to −8.0	< 0.001

Abbreviations: AD‐MSC, adipose‐derived mesenchymal stem cell; CI, confidence interval; UC‐MSC, umbilical cord‐derived mesenchymal stem cell.

### 4.9 Dose–Response Relationships

Three studies systematically evaluated dose–response relationships in adipose‐derived MSC therapy [34, 35].

Suseno et al. (2025) conducted a dose‐finding study comparing 2, 5, and 10 million adipose‐derived MSCs per cm^2^, demonstrating [34]:

- 2 million cells/cm^2^: 28.7% collagen increase; effects persisted 6–8 months
- ≥ 5 million cells/cm^2^: 41.2% collagen increase; effects persisted 9–12 months
- ≥ 10 million cells/cm^2^: No significant additional benefit (43.1% collagen increase) but increased injection site reactions (31% vs. 12% for 5 million, *p* = 0.04)

These findings were corroborated by [35], who reported dose‐dependent improvements in both elasticity and wrinkle reduction with increasing concentrations of adipose‐derived MSC conditioned media [35]. Additionally, [36] demonstrated that repeated applications of stem cell conditioned medium produced enhanced and prolonged clinical outcomes compared to single‐dose administration [36].

Collectively, these studies suggest an optimal therapeutic window exists for adipose‐derived MSC therapies, with doses of approximately 5 million cells/cm^2^ offering maximal efficacy with minimal adverse effects. Doses exceeding this threshold provide diminishing returns while increasing the risk of injection site reactions.

### 4.10 Long‐Term Durability Outcomes

Long‐term follow‐up data (≥ 12 months) were available from 2 studies [13, 35].

Ponnikorn et al. (2026) provided the most extensive long‐term data, with follow‐up at 3 and 5 years post‐treatment in 120 participants [13]. However, as this is a single non‐randomized study with moderate risk of bias, these findings must be interpreted with caution:

- Sustained clinically significant improvement: 78.8% of patients at 3 years
- Sustained clinically significant improvement: 52.9% of patients at 5 years
- Retreatment at 3 years restored improvements to 92.3% of initial response levels.

Lee et al. (2021) reported sustained improvements at 24 weeks (approximately 6 months) but did not provide longer‐term data [35].

No other included studies reported outcomes beyond 12 months. The limited long‐term data suggest that treatment effects are durable for most patients up to 3 years, with a gradual decline by 5 years, and that retreatment can effectively restore benefits. However, these findings are derived from a single study and require confirmation.

### 4.11 Combination Therapy Outcomes

Four studies evaluated combination therapies, primarily stem cell products combined with physical enhancement techniques [34, 35, 36, 39].

#### 4.11.1 Microneedling Combinations

Suseno et al. (2025) reported that microneedling combined with adipose‐derived MSC secretome produced a 28% reduction in wrinkle severity at 6 months compared to 14% with microneedling alone (*p* < 0.01) [34, 36] demonstrated a 3.2‐fold increase in product penetration depth with microneedling‐assisted delivery of MSC‐conditioned medium, resulting in 41.8% improvement in overall skin texture compared to 24.3% with microneedling alone (*p* < 0.05) [36].

#### 4.11.2 Laser Combinations

Lee et al. (2021) demonstrated that fractional CO2 laser followed by MSC‐conditioned media application resulted in superior elasticity improvements compared to laser alone (34% vs. 22%; *p* < 0.05) [35].

#### 4.11.3 Radiofrequency Combinations

Panithaporn et al. (2025) reported enhanced skin quality improvements with red deer umbilical cord MSC‐conditioned media combined with monopolar radiofrequency compared to radiofrequency alone in a small case series (*n* = 10) [39]. However, the non‐human cell source and serious risk of bias limit the generalizability of these findings.

These findings suggest that combination approaches, particularly those integrating stem cell‐based therapies with physical enhancement techniques (microneedling, laser), produce synergistic effects that exceed monotherapy outcomes. Physical enhancement appears to improve product penetration and distribution, potentially explaining the enhanced efficacy.

### 4.12 GRADE Assessment of Primary Outcomes

#### 4.12.1 Skin Elasticity

The certainty of evidence for skin elasticity improvements with stem cell‐based therapies was rated as LOW. This rating reflects: (1) serious risk of bias in several included non‐randomized studies (downgraded one level); (2) inconsistency in outcome measurement methods and follow‐up durations across studies (downgraded one level); and (3) indirectness introduced by the inclusion of studies using non‐human cell sources (avian, plant, red deer) with limited generalizability to human clinical practice (downgraded one level).

#### 4.12.2 Collagen Synthesis

The certainty of evidence for increased collagen synthesis was rated as LOW. This rating reflects: (1) serious risk of bias, particularly in studies lacking blind histological assessment (downgraded one level); (2) inconsistency in collagen measurement techniques and reporting (downgraded one level); and (3) imprecision due to small sample sizes in several studies (downgraded one level).

#### 4.12.3 Wrinkle Reduction

The certainty of evidence for wrinkle reduction was rated as LOW. This rating reflects: (1) serious risk of bias in non‐randomized studies and case series (downgraded one level); (2) inconsistency in wrinkle assessment scales and outcome measures (downgraded one level); and (3) publication bias potentially favoring positive results in this commercially driven field (downgraded one level).

#### 4.12.4 Safety Outcomes

The certainty of evidence for safety was rated as MODERATE. This rating reflects consistent reporting of adverse events across all 9 studies, with no serious safety signals identified. However, the inclusion of non‐human studies introduces some indirectness, and long‐term safety data beyond 5 years remain limited.

## 5 Discussion

This systematic review provides a comprehensive synthesis of primary clinical evidence on stem cell‐based therapies for skin rejuvenation, incorporating data from 9 studies and 847 participants. The findings suggest that mesenchymal stem cell‐based interventions, particularly those derived from adipose tissue and umbilical cord, are associated with clinically meaningful improvements in skin elasticity, collagen synthesis, and wrinkle reduction.

The evidence consistently demonstrates that adipose‐derived MSCs improve skin elasticity by approximately 26%–34% and collagen synthesis by approximately 35%–45% across individual studies. These effect sizes are consistent with those reported in prior systematic reviews of the literature [11].

### 5.1 Differential Efficacy by Stem Cell Source

The discussion of differential efficacy is primarily informed by the head‐to‐head comparison from Ponnikorn et al. (2026) [13]. Given that this is a single study with a moderate risk of bias, the following mechanistic interpretations must be viewed with considerable caution.

The differential efficacy observed across stem cell sources warrants careful mechanistic consideration, though these comparisons must be interpreted with caution, given that only one study provided a direct head‐to‐head comparison [13]. The landmark comparative study by [13] demonstrated that while both umbilical cord‐derived and adipose‐derived MSC exosomes increased fibroblast proliferation and reduced senescence, they exhibited distinct mechanistic profiles [13].

Adipose‐derived exosomes showed higher VEGF content, driving angiogenesis and greater collagen and hyaluronic acid production, making them potentially preferable for patients seeking maximal improvement in skin texture, elasticity, and volume.

Umbilical cord‐derived exosomes were enriched in TGF‐β and PDGF‐BB, demonstrating stronger immunomodulatory activity and more pronounced reduction of inflammatory cytokines, suggesting they may be particularly beneficial for patients with inflammatory skin conditions or significant photoaging.

These findings align with earlier work by [40], who confirmed that umbilical cord‐derived conditioned media had a higher content of effective factors and stimulated greater fibroblast proliferation compared to adipose‐derived counterparts [40]. However, these mechanistic insights are derived from limited comparative data and should be considered hypothesis‐generating rather than conclusive for clinical decision‐making.

### 5.2 Emergence of Exosome‐Based Therapies

The emergence of exosome‐based therapies as cell‐free alternatives represents a paradigm shift in regenerative dermatology. Four studies in this review evaluated exosome‐based approaches [12, 13, 38, 39], demonstrating consistent improvements across multiple skin aging parameters. The investigator‐blind, split‐face non‐inferiority trial by Estupiñan et al. (2025) is particularly noteworthy, directly demonstrating that adipose‐derived MSC exosomes achieve comparable efficacy to platelet‐rich plasma, an established treatment, for photoaged facial skin [12].

Exosome‐based approaches offer several practical advantages over cell‐based therapies, including:

- Avoidance of live cell transplantation, eliminating tumorigenicity concerns
- Reduced immunogenicity, enabling allogeneic use without matching
- Off‐the‐shelf availability, simplifying clinical workflow
- Stable product with defined composition (potentially)
- Avoidance of invasive harvest procedures

The favorable safety profile observed across all exosome studies, with all adverse events being mild, local, and transient, supports the continued development of these approaches.

### 5.3 Interpretation in the Context of Risk of Bias and Heterogeneity

The findings of this review must be interpreted in light of the quality of the included evidence. Among the 5 RCTs, only 2 were rated as low risk of bias [12, 34], with 2 having some concerns [33, 36] and 1 rated as high risk of bias [35]. Among the 4 non‐randomized studies, 1 was rated as moderate risk [13] and 3 as serious risk [37, 38, 39]. A major limitation of this evidence base is the inclusion of three studies with serious risk of bias utilizing non‐human stem cell sources (avian MSCs [37], plant‐derived exosomes [38], and red deer umbilical cord MSCs) [39]. The questionable clinical relevance of these sources for human skin rejuvenation, combined with their high risk of bias, significantly limits their contribution to the evidence base. The findings from these studies should be considered exploratory at best and should not be used to inform clinical practice decisions regarding human stem cell therapies. This distribution of quality ratings, particularly the serious risk of bias in studies with limited generalizability, substantially tempers the certainty of conclusions that can be drawn from this review.

#### 5.3.1 GRADE Assessment

For the primary outcomes of skin elasticity and collagen synthesis, the certainty of evidence is rated as LOW. This rating reflects the serious risk of bias in several included studies, inconsistency in outcome measurement, and indirectness introduced by non‐human cell sources. For safety outcomes, the certainty of evidence is rated as MODERATE due to consistent reporting across studies, though the inclusion of non‐human studies introduces some indirectness.

#### 5.3.2 Limitations of Quantitative Synthesis

While we performed a post hoc restricted meta‐analysis for adipose‐derived MSC RCTs evaluating skin elasticity, several limitations should be acknowledged. The meta‐analysis included only 4 studies with 167 participants, limiting statistical power and precision. Moderate heterogeneity (I^2^ = 68.4%) was observed, attributable to differences in intervention protocols, delivery methods, and combination therapies. The inclusion of one study with high risk of bias [35] may have influenced the pooled estimate. Furthermore, the meta‐analysis could not address other important outcomes (collagen synthesis, wrinkle reduction) due to insufficient homogeneity in outcome measurement. Given these limitations, the meta‐analysis results should be considered exploratory and hypothesis‐generating rather than definitive. We emphasize that our primary conclusions are based on the narrative synthesis of all available evidence, which we believe provides a more comprehensive and cautious interpretation of the current evidence base.

### 5.4 Comprehensive Safety Analysis and Long‐Term Considerations

The favorable safety profile observed across all included studies is reassuring, with 98.4% of adverse events being Grade 1 (mild) and transient. No cases of tumorigenicity, malignant transformation, teratoma formation, or ectopic tissue development were reported in any study, including those with long‐term follow‐up extending to 5 years [13]. However, several important safety considerations warrant discussion.

#### 5.4.1 Tumorigenicity Surveillance

The theoretical risk of tumorigenicity associated with stem cell therapies, particularly those involving live cell transplantation, remains a key concern [41]. The absence of tumorigenicity signals in this review is reassuring but must be interpreted in the context of limited surveillance intervals. The longest follow‐up (5 years) from a single study [13] provides some evidence of medium‐term safety, but longer surveillance (≥ 10 years) is needed to definitively exclude malignancy risk [42]. Carcinogenic surveillance intervals should be standardized in future studies, with recommendations for annual follow‐up for at least 5–10 years post‐treatment.

#### 5.4.2 Immunogenicity

The absence of immune‐mediated rejection reactions, even with allogeneic cell sources, is noteworthy. This may reflect the low immunogenicity of MSCs due to their lack of MHC‐II expression and immunomodulatory properties [43]. However, immunogenicity assessment in the included studies was limited to clinical observation of adverse events rather than systematic immune monitoring (e.g., HLA antibody testing). Future studies should incorporate systematic immunogenicity assessment to fully characterize the immune response to allogeneic stem cell products [44].

#### 5.4.3 Infection Risk

No infections were reported in any included study, reflecting the low infection risk associated with these minimally invasive procedures. However, as with any injectable therapy, the risk of infection exists and should be mitigated through strict aseptic technique and appropriate patient selection [45].

#### 5.4.4 Dose‐Safety Relationship

The dose–response data from Suseno et al. (2025) [34] demonstrate a clear dose‐safety relationship, with higher cell doses (≥ 10 million cells/cm^2^) associated with increased injection site reactions (31% vs. 12%; *p* = 0.04). This finding highlights the importance of dose optimization to balance efficacy and safety.

#### 5.4.5 Limitations ofh3 Current Safety Data

Several limitations of the current safety evidence base should be acknowledged. First, the short follow‐up duration in most studies (3–6 months) precludes assessment of long‐term safety outcomes. Second, the small sample sizes in several studies limit the ability to detect rare adverse events. Third, the lack of standardized safety reporting and systematic adverse event monitoring may have resulted in underreporting of mild events. Fourth, the inclusion of studies using non‐human cell sources with limited safety data introduces additional uncertainty [41, 42].

#### 5.4.6 Recommendations for Safety Surveillance

Based on these considerations, we recommend that future studies: (1) implement standardized safety reporting using CTCAE or similar grading systems; (2) include systematic immunogenicity assessment for allogeneic products; (3) incorporate long‐term follow‐up (≥ 5 years) with standardized surveillance intervals; (4) establish registries to monitor real‐world safety outcomes; and (5) develop consensus guidelines for safety monitoring in stem cell‐based skin rejuvenation trials [41, 44].

### 5.5 Regulatory Considerations

The regulatory landscape for stem cell‐based products varies considerably by jurisdiction and product type, with important implications for clinical translation and product claims.

#### 5.5.1 Cell‐Based Therapies (Live MSCs)

In the United States, live MSC products are regulated as biologics by the FDA's Center for Biologics Evaluation and Research (CBER) and typically require Investigational New Drug (IND) applications and Biologics License Applications (BLAs) for marketing [46]. In the European Union, these products are classified as Advanced Therapy Medicinal Products (ATMPs) and require centralized marketing authorization from the European Medicines Agency (EMA) [47]. The regulatory pathway is rigorous, requiring extensive preclinical safety data, Good Manufacturing Practice (GMP) compliance, and controlled clinical trials demonstrating safety and efficacy [48]. The studies reviewed here that utilized live MSCs all reported compliance with relevant regulatory standards.

#### 5.5.2 Cell‐Free Products (Exosomes, Conditioned Media, Secretome)

The regulatory status of cell‐free products is more complex and evolving. In many jurisdictions, these products may be regulated as biologics, medical devices, or cosmetics depending on their intended use and manufacturing process [49]. Plant‐derived exosomes, such as the rose stem cell‐derived exosomes evaluated in one included study, are typically registered as cosmetic products in most jurisdictions. This distinction is clinically relevant: Cosmetic products are subject to less rigorous regulatory oversight than biologics and cannot legally make therapeutic claims. Clinicians and patients should be aware that plant‐derived exosome products marketed as cosmetics may not have undergone the same level of safety and efficacy testing as regulated biologics [50].

#### 5.5.3 Alignmhent With Product Claims

The studies included in this review vary in their alignment of product claims with regulatory status. Human‐derived MSC products were uniformly studied as investigational biologics with appropriate regulatory oversight. However, the regulatory status of some products, particularly plant‐derived exosomes, was not clearly specified. Future research should explicitly report the regulatory classification of investigational products and ensure that product claims align with regulatory status. This is particularly important given the proliferation of commercially marketed stem cell and exosome products for skin rejuvenation, many of which may not meet regulatory standards for safety and efficacy [48, 50].

#### 5.5.4 Implications for Clinical Practice

Clinicians should be aware of the regulatory status of products they use or recommend and should prioritize products that have undergone rigorous regulatory review. Patients should be counseled that regulatory oversight varies by product type, with human‐derived biologics subject to more rigorous oversight than cosmetic products. The absence of serious safety events in this review should not be interpreted as evidence that all products on the market are safe; rather, the safety data are limited to products studied in controlled clinical trials with appropriate regulatory oversight [48, 49].

#### 5.5.5 Future Regulatory Developments

The rapid evolution of stem cell and exosome technologies presents challenges for regulators. We anticipate that regulatory frameworks will continue to evolve, with increased scrutiny of cell‐free products and development of product‐specific standards. The field would benefit from international harmonization of regulatory standards and development of clear guidance for the clinical development of stem cell‐based products for skin rejuvenation [47, 49].

### 5.6 Limitations of Non‐Human Stem Cell Source Evidence

A significant limitation of the current evidence base is the inclusion of three studies utilizing non‐human cell sources (avian MSCs, plant‐derived exosomes, and red deer umbilical cord MSCs). These studies, while providing interesting preliminary data, operate under fundamentally different regulatory, immunological, and translational frameworks compared to human‐derived products [51]. Xenogeneic products carry distinct immunogenicity concerns, including potential reactions to α‐gal epitopes and other species‐specific proteins, which are not directly comparable to human‐derived products [52]. Plant‐derived exosomes, e.g., are typically registered as cosmetic products rather than biologics, and while they exhibit low immunogenicity and favorable biocompatibility in preclinical studies, their mechanisms of action may differ substantially from mammalian stem cell products [53].

The inclusion of these studies in our synthesis, while appropriate for completeness, introduces substantial heterogeneity and limits the generalizability of our findings. We have therefore presented these results separately (Section 4.1.5) and excluded them from our primary conclusions regarding clinical efficacy and safety. Future systematic reviews should consider establishing a priori criteria for the inclusion of non‐human studies, potentially restricting primary syntheses to human‐derived products with validation in human subjects. The field would benefit from standardized guidelines for the preclinical and clinical evaluation of xenogeneic and plant‐derived products to ensure appropriate regulatory oversight and patient safety [51, 53].

### 5.7 Cost‐Effectiveness Considerations

Although not directly evaluated in the included studies, cost‐effectiveness is an important consideration for clinical translation. Current stem cell‐based therapies for skin rejuvenation are typically expensive, with costs ranging from several hundred to several thousand dollars per treatment session, depending on the product, delivery method, and geographic location. Cell‐free products (exosomes, conditioned media) may offer cost advantages over live cell therapies due to simplified manufacturing, storage, and distribution logistics [54]. However, the lack of head‐to‐head cost‐effectiveness analyses in the literature represents a significant gap. Future research should incorporate health economic evaluations to inform reimbursement decisions and patient access [55]. Additionally, the durability of treatment effects is a critical determinant of cost‐effectiveness; longer‐lasting effects may justify higher upfront costs compared to repeated conventional treatments [54, 55].

## 6 Conclusion

This systematic review synthesizes the available evidence from human‐derived studies, which is of low to moderate certainty, suggesting that stem cell‐based therapies, particularly adipose‐derived and umbilical cord‐derived mesenchymal stem cells and their exosomes, may represent effective and safe interventions for skin rejuvenation. Evidence from three non‐human studies (avian MSCs, plant‐derived exosomes, and red deer umbilical cord MSCs) is presented separately as exploratory and requires validation in human‐derived cell studies before clinical translation.

Findings from individual studies indicate adipose‐derived MSCs are associated with improvements in elasticity and collagen synthesis, while umbilical cord‐derived cells may offer enhanced anti‐inflammatory effects based on a single comparative study. The emergence of exosome‐based therapies is promising and may address some safety concerns.

However, significant limitations in the current evidence base must be acknowledged, including heterogeneity in study designs, lack of standardized protocols, and underrepresentation of diverse populations. Furthermore, the low to moderate certainty of evidence and potential for bias in included studies mean that effect estimates may change as higher‐quality research becomes available. The inclusion of studies using non‐human cell sources with a serious risk of bias further limits the generalizability of findings. Future research should prioritize large, multicenter randomized controlled trials with standardized methodologies, long‐term follow‐up, and cost‐effectiveness analyses. With continued advances in stem cell technology, these therapies hold promise for transforming dermatological practice by addressing the root causes of skin aging rather than merely its superficial manifestations.

## Funding

The authors have nothing to report.

## Conflicts of Interest

The authors declare no conflicts of interest.

## Supporting information


**Table S1:** Meta‐Analysis of Adipose‐Derived MSC Therapies for Skin Elasticity.

## Data Availability

The data that support the findings of this study are available from the corresponding author upon reasonable request.
