# Supplementary material for: Stem Cell‐Based Therapies for Skin Rejuvenation: A Systematic Review of Clinical Efficacy, Safety, and Underlying Mechanisms
Source: J Cosmet Dermatol. 2026 Sep 9;25(9):e71186. doi: 10.1111/jocd.71186 (PMC13555514; doi:10.1111/jocd.71186)
Supplement: Supplementary file 1 [file JOCD-25-e71186-s001.docx]

**Supplementary Table S1: Meta-Analysis of Adipose-Derived MSC Therapies for Skin Elasticity**

| **Study** | **n** | **Intervention** | **Effect Size**  **(95% CI)** | **Weight (%)** |
| --- | --- | --- | --- | --- |
| **Estupiñan et al. 2025** | 52 | AD-MSC exosomes vs. PRP | 31.0% (25.8-36.2) | 28.4 |
| **Suseno et al. 2025** | 60 | AD-MSC secretome + MN vs. MN alone | 26.0% (21.0-31.0) | 27.6 |
| **Lee et al. 2021** | 30 | AD-MSC CM + CO2 laser vs. laser alone | 34.0% (28.6-39.4) | 24.1 |
| **Lee et al. 2014** | 25 | AD-MSC CM + MN vs. MN alone | 34.5% (28.4-40.6) | 19.9 |
| **Pooled (random effects)** | **167** |  | **30.7% (26.4-35.0)** | **100%** |

*Abbreviations: AD-MSC, adipose-derived mesenchymal stem cell; CM, conditioned media; MN, microneedling; PRP, platelet-rich plasma; CI, confidence interval.*

*Heterogeneity: I² = 68.4%; τ² = 0.012; Chi² = 9.48, df = 3, p = 0.02*
